# Supplementary material for: Robust Room-Temperature Quantum Spin Hall Effect in Methyl-functionalized InBi honeycomb film
Source: Sci Rep. 2016 Mar 21;6:23242. doi: 10.1038/srep23242 (PMC4800414; doi:10.1038/srep23242)
Supplement: Supplementary Information [file srep23242-s1.pdf]

Supplementary Materials for

Robust Room-Temperature Quantum Spin Hall Effect in Methyl-functionalized InBi honeycomb film

Sheng-shi Li,[a, b] Wei-xiao Ji,[a] Chang-wen Zhang*,[a] Shu-jun Hu,[b] and Ping Li,[a] Pei-ji Wang [a], Bao-min Zhang, [a] and Chong-long Cao [a]

*Correspondence author E-mail: zhchwsd@163.com

This file includes:

Figure S1 to S3

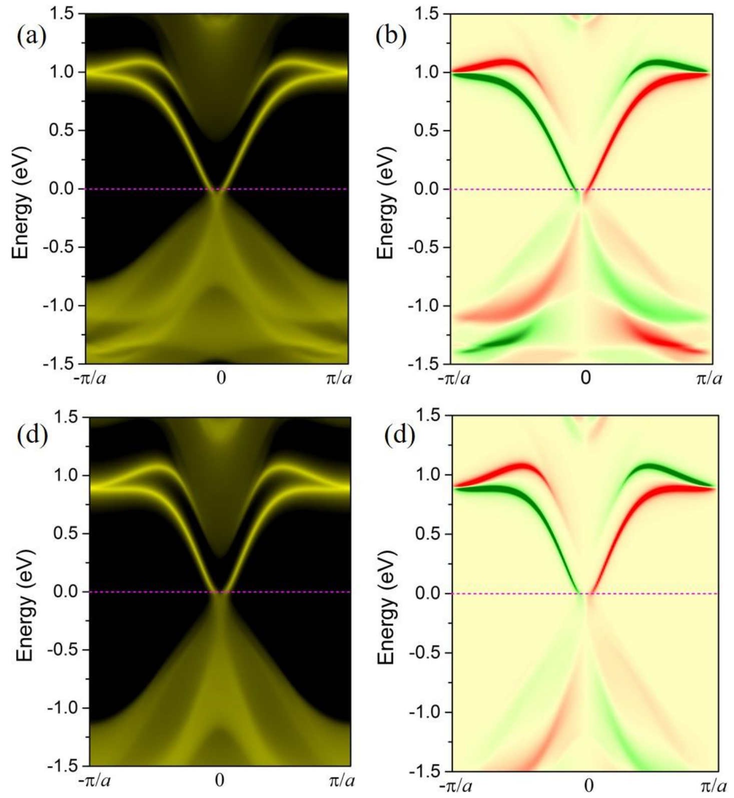

Figure S1 The calculated semi-infinite edge states of total (a) and spin (b) for InBiCH3 film with 7% tensile strain. (c) and (d) are corresponding edge states with electric field of 0.5V/ Å.

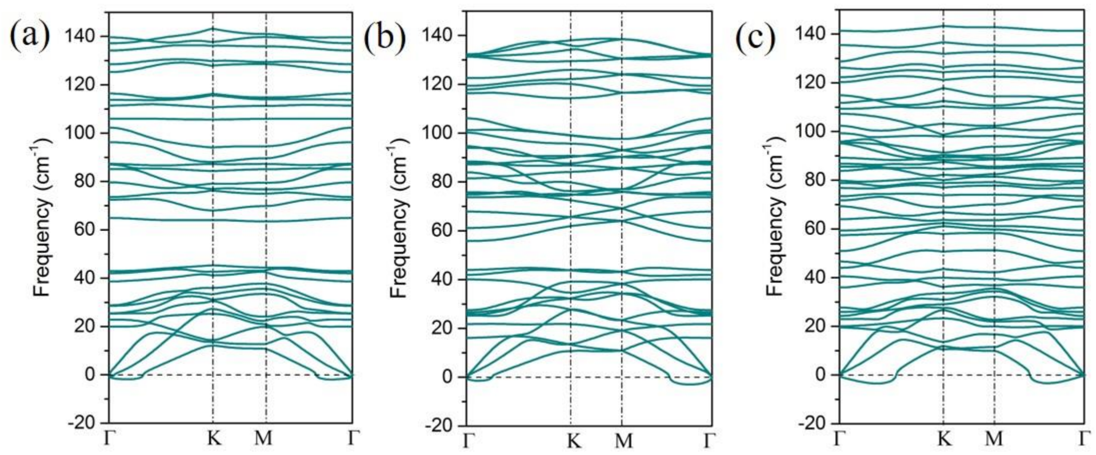

Figure S2 The calculated phonon spectra along the high-symmetric points in the BZ for (a) 0.25, (b) 0.5 and (c) 0.75 monolayer coverage.

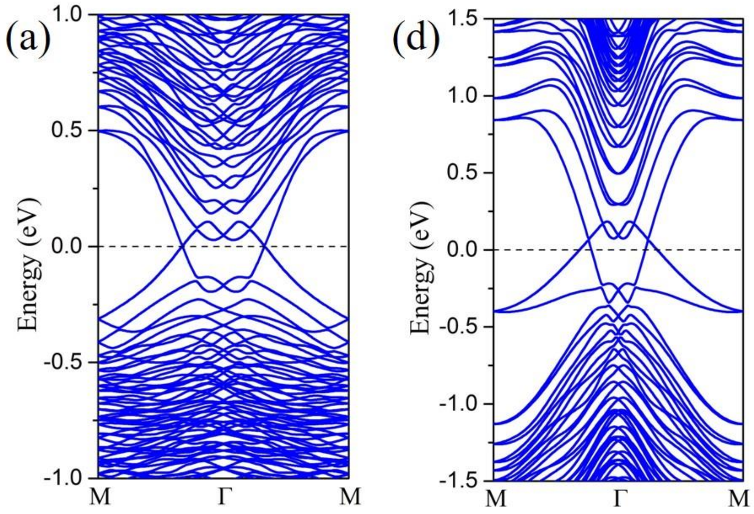

Figure S3 Band structures of InBiCH3 for a zigzag ribbon. (a) and (b) are the representative of 0.25 monolayer and $InBiCH_3$@2×2BN HBL, respectively. The Fermi level is set to zero.
